# Neoadjuvant chemotherapy in locally advanced duodenal adenocarcinoma

**DOI:** 10.3332/ecancer.2018.816

**Published:** 2018-03-05

**Authors:** Carlos Velandia, Rafael Delgado Morales, Carlos Coello, Armando Gil Mendoza, Gabriel Pérez, Emperatriz Aguero

**Affiliations:** 1 Surgical Oncology IOLR, Digestive Pathology Service, Instituto de Oncologia ‘Dr Luis Razetti’ (IOLR), Caracas 1010, Venezuela; 2 Digestive Tract Service IOLR, Digestive Pathology Service, Instituto de Oncologia ‘Dr Luis Razetti’ (IOLR), Caracas 1010, Venezuela; 3 Anatomic Pathology Service, Digestive Pathology Service, Instituto de Oncologia ‘Dr Luis Razetti’ (IOLR), Caracas 1010, Venezuela

**Keywords:** duodenal adenocarcinoma, neoadjuvant therapy

## Abstract

Duodenal adenocarcinoma (ADC) represents only 0.3% of gastrointestinal neoplasms. With the frequency being higher between the ages of 40 and 60, it is predominantly located in the second part of the duodenum and around the periampullary region. Symptoms are nonspecific, so the majority of patients present with advanced disease. Neoadjuvant chemotherapy is a therapeutic option that has not been well studied. The global literature includes only isolated reports on this subject. This is why we are presenting the following case: a 60-year-old female patient with a locally advanced, inoperable duodenal ADC received neoadjuvant chemotherapy. Having presented a favourable response as observed in the post-neoadjuvant extension studies, a pancreaticoduodenectomy was performed without any perioperative complications and with satisfactory progress. The final biopsy reported a complete pathological response. After being monitored for 34 months, the patient was free from locoregional and distant metastatic disease. During the last weeks of monitoring, she developed a second primary breast tumour, which has been corroborated by immunohistochemistry.

## Introduction

Adenocarcinoma is the most common malignant tumour of the digestive tract. It is a major cause of morbidity and mortality around the world. The small intestine comprises 75% of the entire length of the gastrointestinal tract and 90% of its mucosal surface. However, it is an uncommon place to develop cancer; fewer than 5% of gastrointestinal cancers are found here. The main histological types of cancer in the small intestine are adenocarcinoma, neuroendocrine tumours, lymphomas and gastrointestinal stromal tumours. The first two histological types comprise 80% of cases with a very similar ratio. In the United States of America, the annual incidence is 5,300 cases with a mortality rate of 1,100 deaths per year. These data have remained stable during the last 15 years; in Europe, according to EUROCARE, there are 3,600 cases each year with an estimated incidence of 5.7 cases per 1 million people [1, 2]. In Venezuela, there were 46 deaths due to malignant tumours of the duodenum in 2012 [3].

The main therapeutic option is surgery. The type of surgical resection depends on the size and location of the tumour. Pancreaticoduodenectomies or segmental resections are the most common procedures [4, 5]. Due to the low incidence of this type of disease, there is no standardised form of chemotherapy (CT). There are only some isolated published reports. In many cases, the use of these reports as adjuvant options relies on selection criteria, as well as drawings of established treatments for colorectal cancer. In cases of lesions in the second part of the duodenum that are adjacent to the papilla, these are treated similarly to pancreatic adenocarcinoma [6, 7].

As a result, CT is commonly used in duodenal adenocarcinomas (ADCs). Neoadjuvant CT is one option. Current scientific evidence that supports this treatment method is based on retrospective studies or anecdotal experiences. There are very few published cases. In view of this fact, we are presenting the case of a female patient with a locally advanced duodenal ADC who received neoadjuvant CT with a positive result.

## Clinical Case

A 60-year-old female patient reported the onset of her present illness roughly 6 months ago. Her illness was characterised by abdominal pain in the epigastrium without irradiation but with a moderate intensity that was partially relieved by ingesting NSAIDs. During the same time she lost about 20 kg. Presenting with these symptoms, the patient visited a physician who suggested an abdominal ultrasound. This exam reported a tumour on the head of the pancreas measuring 72.9 × 59.9 with irregular edges. Later, an upper gastrointestinal endoscopy showed an infiltrating duodenal tumour in the second part of the duodenum with gastroduodenal extrinsic compression. A sample of the duodenal tumour was taken, which showed moderate chronic duodenitis and reactive lymphoid hyperplasia. With this clinical picture in mind, the patient was referred to the Instituto de Oncología Dr ‘Luis Razetti’ (IOLR).

**Figure 1.**
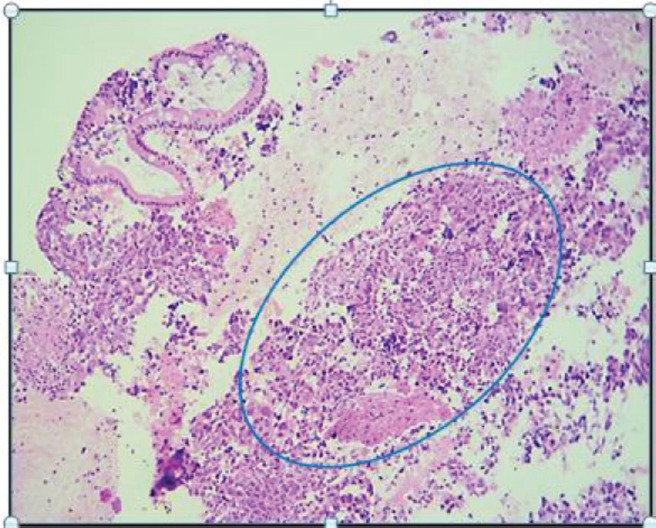
H & E-stained histological preparation. Tumoural lesion with unorganised, diffuse growth, where normal glandular architecture is lost, and nuclear atypia is present.

Her family history showed that she had a sister who had breast cancer at the age of 40. She said that she had not had any significant pathological or surgical history. The physical examination showed that the patient was in good general health and afebrile with an Eastern Cooperative Oncology Group (ECOG) Performance Status of 0. Her neck showed no adenopathies; the abdomen was soft and palpable without pain and there was no sign of tumours; the rectal examination showed a lesion-free normotonic sphincter. At the IOLR Digestive Tract Service, an upper gastrointestinal endoscopic ultrasound was requested. It showed a mucosa with an infiltrative appearance in the second and third parts of the duodenum. It also showed a conglomerate retroperitoneal lymph node involving the second and third parts of the duodenum. In addition, there were numerous adenopathies in the peri-aortic/caval region and in the hepatic hilum. The pancreas showed moderate adipose infiltration without any tumour. The inferior vena cava showed compression from the designated lesion without infiltration. A biopsy from the second and third part of the duodenum was taken and sent to create a cell block, the histopathological reports of which showed a carcinoma that was poorly differentiated from the duodenum (Figure 1). An immunohistochemistry was requested and this showed a positive immunoreactivity for Cytokeratin AE1/3 and a negative immunoreactivity to the common leukocyte antigen, Synaptophysin, Cytokeratin 20, chromogranin and CDX2. Ki-67 was 80%. The conclusion was a poorly differentiated duodenal ADC.

**Figure 2.**
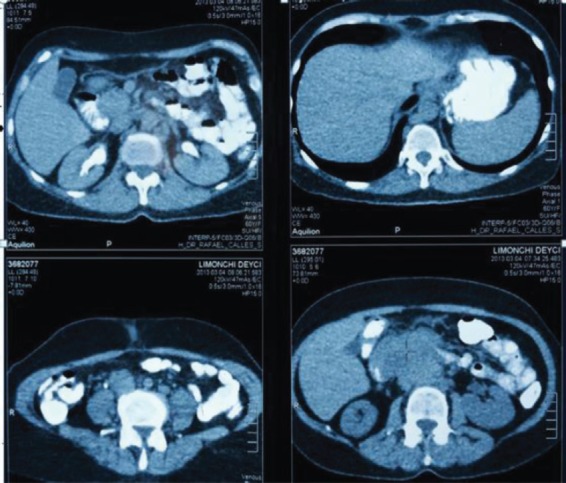
Abdominopelvic CT with oral and intravenous contrast during the elimination phase, axial sections.

**Figure 3.**
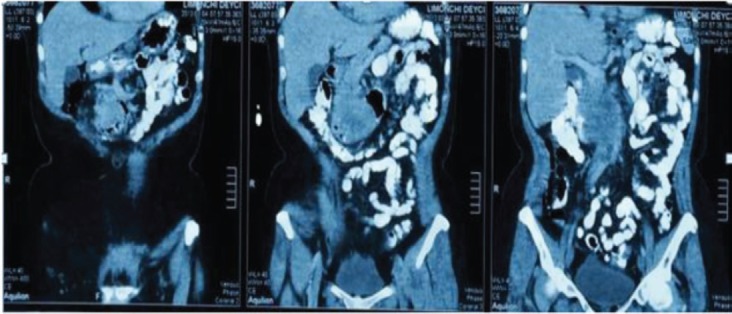
Abdominopelvic CT with oral and intravenous contrast in the elimination phase, coronal sections.

Extension studies were made: a chest CT, where there was no evidence of any images with metastasis, and an abdominal and pelvic CT that showed hypodense images, irregular edges adjacent to the second and third parts of the duodenum, and irregular enhancement after the administration of contrast. In addition, a conglomerate lymph node was found in the interaortocaval region without any liver metastasis; CA 19.9 and carcinoembryonic antigen levels were within the reference ranges (Figures 2 and 3). In light of the various findings above, the Digestive Tract Service at the IOLR considered the case as a locally advanced, inoperable duodenal ADC. Consequently, we discussed the case with the Internal Medical Oncology Service and decided to start neoadjuvant CT as follows: oxaliplatin and capecitabine for six cycles, at intervals of 21 days, administered completely without toxicity.

After the neoadjuvant CT, the following restaging studies took place: (1) Abdominal and pelvic CT: solid duodenal SOL measuring 4 mm with highlighted enhancement after administering intravenous contrast but no metastatic lesions; para-aortic lymph nodes measuring less than 5 mm. (2) Chest CT: no metastatic lesions. (3) Upper gastrointestinal endoscopy: duodenal bulb without lesions progressing to the third part of the duodenum where there was evidence of completely normal papilla; no infiltration or compression of the mucosa was seen. There were no lesions in the duodenum. (4) Upper gastrointestinal endoscopic ultrasound: a nonconcentric, hypoechoic lesion with imprecise edges between the second and third parts of the duodenum, with minimum extramural extension toward the distal end of the lesion; local inflammatory changes with perilesional adenopathies and in the hepatic hilum that appear to be benign; pancreas with moderate adipose infiltration not showing any tumours; inflammatory changes around the vena cava; and no evidence of extrinsic compression by a primary lesion as evidenced in the previous study.

In view of the positive response observed in the extension studies, a pancreaticoduodenectomy was planned. The intraoperative findings were a lesion-free peritoneal cavity and liver. In the second and third parts of the duodenum, a diffuse thickening with loose adhesions to the retroperitoneum and without any interaortocaval adenopathies was palpable. The pancreaticoduodenectomy was performed without any immediate or late complications. The patient was discharged on the fifth postoperative day. The final biopsy was a sample from the pancreaticoduodenectomy: the duodenum showed moderate chronic infiltration with areas of ulceration and no evidence of neoplasm; the head of the pancreas showed mild chronic inflammation and foci of interstitial fibrosis, 12 lymph nodes with nonspecific reactive follicular hyperplasia and adipose infiltration, but these were negative for malignancy; the gallbladder showed chronic cholecystitis, cholesterolosis and three lymph nodes with nonspecific reactive paracortical hyperplasia in the hepatic pedicle without any evidence of neoplasm.

Six weeks after the surgery, the case was discussed again with the Internal Medical Oncology Service. It was decided together that the patient would be monitored via physical examinations every 3 months for 2 years, and then every 6 months until the fifth year. She would also undergo imaging and endoscopic studies on an annual basis. At present, the patient has been free from locoregional or distant metastatic disease for 34 months. Recently, she developed a right breast ductal adenocarcinoma, with immunohistochemistry ER + PR + HER2 − Ki−67 15% T1NxM0. She is waiting for a surgical appointment at the IOLR’s Breast Care Service.

## Discussion

Duodenal ADC was first described in 1746 by Hamburger. It represents only 0.3% of gastrointestinal neoplasms and occurs in 45% of cases in the third and fourth parts of the duodenum. It is found most frequently in males aged 40–60. Furthermore, duodenal ADC is still a fatal disease, even though it can be removed surgically [8]. Occurrence increases in patients with Lynch syndrome, which is considered responsible for 5–10% of the cases, familial adenomatous polyposis, which is usually associated with numerous duodenal polyps, and Peutz–Jeghers syndrome where the occurrence of adenocarcinoma of the small intestine is also higher than in the general population [9, 10].

Duodenal ADCs usually affect a short segment. They feature progressive annular growth and often lead to delayed stenosis and ulceration. The symptoms are nonspecific and resemble those of a duodenal ulcer. For this reason, most patients present with an advanced disease. The staging of this type of lesion is done through studies that assess locoregional extension, such as an endoscopic ultrasound; and those that assess locoregional and distant metastatic extensions, such as a tomography of the chest, abdomen and pelvis [11].

The treatment of choice for duodenal malignant neoplasms is surgery. It is defined as a resection of the entire tumour with lesion-free margins in the absence of metastatic disease. This can be carried out either through a pancreaticoduodenectomy or via a segmental duodenal resection. The type of pancreaticoduodenectomy that is usually indicated for tumours of the first and second parts of the duodenum, or the duodenal resection for distal tumours to the superior mesenteric vein, is usually performed for healing purposes. In cases when surgery is not possible, or if the patient condition contraindicates surgery, there are intestinal bypass or CT procedures that act as palliative treatment [12].

According to the literature, at the time of diagnosis, only between 40% and 70% of patients were able to receive curative surgical treatment. Overall survival after 5 years is 25–35%. This number is directly related to lymphatic compromise, tumour location, transmural invasion and histologic classification. A much poorer prognosis is noted in those who are not candidates for surgery [13–16]. Our patient presented with a bulky lesion in the second and third parts of the duodenum that displaced the inferior vena cava without infiltrating it, and a conglomerate lymph node in the interaortocaval region. In the first assessment, these were considered inoperable. Consequently, an IOLR multidisciplinary meeting recommended neoadjuvant CT.

The role of neoadjuvant therapy for patients with unresectable, locally advanced duodenal ADCs has not been well studied. It is unknown whether this represents a potential rescue treatment for a selected subset of patients with this type of cancer. In 2015, the National Cancer Institute in the United States established palliative measures, CT or chemoradiotherapy (CRT) as recommended treatments for patients who were not candidates for surgical resection, along with a monitoring of any complications. It also considered that there were new treatment methods being studied that essentially consisted of combinations of new drugs. However, radical surgery was not recommended.

The first recorded use of neoadjuvant CT was in 1994, when Coia *et al* [19] conducted a study of preoperative CT/RT in patients with ADC in the head of the pancreas and in the second part of the duodenum. Four patients had tumours in the duodenum and, as a result, it was found that all patients had a complete pathological response after radical surgery. It was concluded that, although the oncological results were excellent, it was not possible to recommend this type of treatment due to the small number of reported patients [17]. In another newer clinical trial run at Duke University by Kelsey *et al* [23], the value of CRT was studied in patients with duodenal ADC compared to surgery alone. Each study group comprised 16 patients. Out of the 16 patients who received CRT, 11 were preoperatory. Two of the patients (18%) showed a complete pathological response. Out of all the resected patients, none of them presented with nodal disease. The conclusion of this study was that the group of patients receiving CRT had a better overall survival rate and were disease-free. It was observed that 11 of the 16 patients received neoadjuvant CT with a good response rate [18].

Currently, no selection criteria have been approved for patients with duodenal cancer receiving neoadjuvant treatment. The choice of treatment depends on the preference of the medical oncologist. Such criteria are often similar to those used in colorectal or pancreatic carcinoma [19, 20]. Our patient presented with a disease that extended to the retroperitoneum, which classified her as inoperable. After receiving neoadjuvant CT, the response from the extension studies was positive. A pancreaticoduodenectomy was then performed resulting in a histopathological report showing a complete pathological response (pCR). The only neoadjuvant CT report on a locally advanced, inoperable duodenal ADC was published in 2012 by Onkendi *et al* [4]. After almost 20 years of revisions, the study found ten patients who were inoperable for various reasons. They received either CT or CRT. It was observed that nine of them were able to have surgery (R0), and two of them showed pCR, which is similar to our described case [4, 21, 22].

Cancer treatment has evolved significantly in the last decades. It is becoming more and more specific for each patient. Consensus meetings or those attended by large groups of experts make recommendations based on highly statistical studies, but there are cases that escape conventionally published standards [23]. At present, cancer treatment is based on customised therapy, i.e., each individual with a type of cancer is unique. Molecular and genetic profiling brings us even closer to treating our patients in this way [24]. In our case with inoperable duodenal ADC, neoadjuvant CT was indicated. This made the surgical procedure possible and pCR was observed. This treatment does not fall under current recommendations, however, in this case, by individualising the clinical data of the response to therapy, the radical surgery option was chosen. It produced excellent oncological results and an overall survival rate of more than 34 months disease-free.

## Conclusion

We can conclude that in patients with duodenal ADC, neoadjuvant CT is a therapeutic option for a subset of inoperable patients as it can decrease the tumour size and lead to surgical resection with negative oncological margins.
